# Dental Department of the Meharry Medical College

**Published:** 1888-11

**Authors:** 


Dental Department of the Meharry Medical College. —We learn from the Nashville *Democrat* of October 24, 1888, that the corner-stone of this college was laid in that city on the 23d ult., Bishop Walden of the M. E. Church officiating. When this building is completed, it will be the only fully-equipped dental college in the world devoted to the colored race.

